# Survival of Enveloped and Non-Enveloped Viruses on Inanimate Surfaces

**DOI:** 10.1264/jsme2.ME14145

**Published:** 2015-04-03

**Authors:** Swan Firquet, Sophie Beaujard, Pierre-Emmanuel Lobert, Famara Sané, Delphine Caloone, Daniel Izard, Didier Hober

**Affiliations:** 1 Université Lille 2, Faculté de Médecine, CHRU LilleLaboratoire de Virologie EA3610, Lille 59037France; 2 CHRU Lille Laboratoire de BactériologieLille 59037France

**Keywords:** coxsackievirus B4, influenza A virus, minute virus of mice, herpes simplex type 1, persistence

## Abstract

In the present study, we evaluated the viability of non-enveloped viruses, minute virus of mice (MVM) and coxsackievirus B4 (CVB4), and enveloped-viruses, influenza A virus (H1N1) and herpes simplex virus type 1 (HSV-1), on surfaces. We also investigated the impact of the initial concentration of proteins and sodium chloride on the persistence of infectious CVB4 on surfaces. Viral suspensions (>10^4.5^ TCID_50_) were applied to petri dish lids and dried under the air flow of a biosafety cabinet. The recovered viral preparations were titered on appropriate cell lines. Enveloped viruses persisted for less than 5 days while CVB4 and MVM persisted for weeks. However, repetitive cycles of drying and resuspension had a stronger virucidal effect on CVB4 than on H1N1 and HSV-1. These repetitive cycles had no effect on the infectious titer of MVM. When exposed to drying, the initial concentrations of bovine serum albumin (from 0 to 90 mg mL^−1^), fetal calf serum (from 0 to 100%), and sodium chloride (from 0 to 300 mg mL^−1^) affected the viability of CVB4. CVB4 was more likely to be inactivated by drying in a protein-rich medium, whereas the impact of drying was reduced in the presence of sodium chloride. The results of the present study demonstrated that the resistance of viruses to drying, as suggested by iterative drying, was not due to the heterogeneity of viral subpopulations, but was influenced by media compositions and component concentrations, as illustrated in the model of CVB4.

Non-enveloped viruses, such as coxsackieviruses, rotavirus, or poliovirus, can survive for extended periods on surfaces (9, 10), while enveloped viruses, including H1N1 and human coronaviruses, remain infectious on surfaces after several days (6). The persistence of dried viruses is affected by various environmental conditions and factors such as heat, moisture, pH, and the type of surface (12, 15). Furthermore, the compositions of media may also influence the persistence of viruses. The impact of drying on viral persistence has been evaluated in previous studies using viruses typically prepared in standardized media, including a cell culture medium supplemented or not with fetal calf serum (FCS) (4, 19). However, these media are not representative of clinical situations because viruses have been detected in protein-rich media, such as serum, and protein-poor media, including water (11, 17).

In the present study, the persistence of two enveloped viruses, influenza virus type A (H1N1) and herpes simplex virus type 1 (HSV-1), and two non-enveloped viruses, minute mouse virus (MVM) and coxsackievirus B4 (CVB4), was investigated. These viruses are relevant models for assessing viral persistence. MVM is a member of the *Parvoviridae* family, which is known to be resistant to desiccation (9), and the other viruses have been implicated in nosocomial infections (5). Furthermore, infectious titers higher than 10^6^ TCID_50_ mL^−1^ can be obtained for each virus *in vitro*.

In addition, components of virus-containing media may affect viral persistence. Therefore, the influence of concentrations of common media components (fetal bovine serum [FCS], bovine serum albumin [BSA] and sodium chloride [NaCl]) on the persistence of CVB4 was also investigated.

## Materials and Methods

### Viruses and cell lines

CVB4 E2 is a strain provided by Ji-Won Yoon (Calgary, Alberta, Canada) (8, 13). HSV-1 (ATCC VR-260), CVB4, MVM (ATCC VR-1346), and H1N1 A/PR/8/34 (ATCC VR-1469) were propagated on Vero (ATCC CCL-81) cells, Hep-2 (ATCC CCL-23) cells, A9 (EACC N° 85011426) cells, and MDCK (NBL2) (ATCC CCL-34) cells, respectively. Infected A9 cells cultured in supplemented DMEM and supplemented MEM (Invitrogen, France) were used to culture Vero and Hep-2 cells. MEM and DMEM media were supplemented with 2% fetal bovine serum (FBS), 1% non-essential amino acids, and 1% L-glutamine at 37°C and incubated in a 5% CO_2_ atmosphere. Infected MDCK cells were cultured in MEM at 35°C in a 5% CO_2_ atmosphere. When cell lysis reached at least 75%, the inoculated flasks were scratched, freeze-thawed three times, and centrifuged at 2,000×*g* for 10 min at 4°C. The supernatant was aseptically aliquoted and stored in a −80°C freezer. The viral titer was determined by limiting a dilution assay to 50% of the tissue culture infection dose by the method of Spearman-Kärber method and expressed as log_10_ TCID_50_ 50 μL^−1^ (7).

### RT real-time PCR

CVB4E2-positive strand RNA was quantitated by two-step quantitative RT-PCR as described previously (15). Briefly, total RNA was extracted with the RNeasy minikit (Qiagen, Valencia, California). Total RNA was retro-transcribed to cDNA using the Affinityscript QPCR cDNA synthesis kit and a specific reverse primer at 42°C for 15 min. Positive strand-specific RT was quantitated by the brilliant II QPCR kit (Agilent Technologies Stratagene) under universal cycle conditions (10 min at 95°C, 40 cycles of 30 s at 60°C) on a Mx3000p (Stratagene). Primers were located within the enterovirus 5′-nontranslated region, which was highly conserved among enterovirus serotypes: CVB4 forward (5′-CCCTGAAT GGGGCTAATC) and CVB4 reverse (5′-ATTGTCACCATA AGCAGCCA). The sequence of the CVB4 probe was 5′-VICAACCGACTACTTTGGGTGTCCGTGTTT- TAMRA (Applied Biosystems). Negative controls were performed by RT-PCR without the reverse transcriptase enzyme or without DNA. Primers and probe pairs were designed with PrimerExpress software, and data were analyzed with Sequence Detector version 1.6.3 (both from Perkin- Elmer, Boston, Massachusetts). Results were expressed as the cycle threshold (Ct)

### Viral persistence

Fifty microliters (10 μL for iterative drying) of the virus inocula were applied to the middle of each petri dish lid (35-mm diameter [Falcon]), and dried under the air flow of a class II biological safety cabinet at room temperature. The air flow speed was 0.4 m s^−1^ and the relative humidity was measured using a relative humidity meter (Temperature and Humidity Data logger, IHM). Medium (1 mL) was added to recover dried virus inocula. The infectious titers of harvested fluids were then determined and the results expressed as described above.

### Statistical analysis

Statistical analyses of the results were performed by the Mann–Whitney U test using Graphpad Prism version 5.00 (Graphpad Software, San Diego, USA) when appropriate. Differences were considered to be significant when *P* <0.05.

## Results

### Infectious level of dried inocula

Fifty microliters of clarified culture supernatant fluids from cells infected with CVB4, MVM, H1N1, or HSV-1 were applied to petri dish lid surfaces and then dried under the airflow of a class II biological safety cabinet at room temperature (20 ± 2°C). The dried inocula were recovered and titrations were performed as described in the Materials and Methods section.

**Fig. 1:**
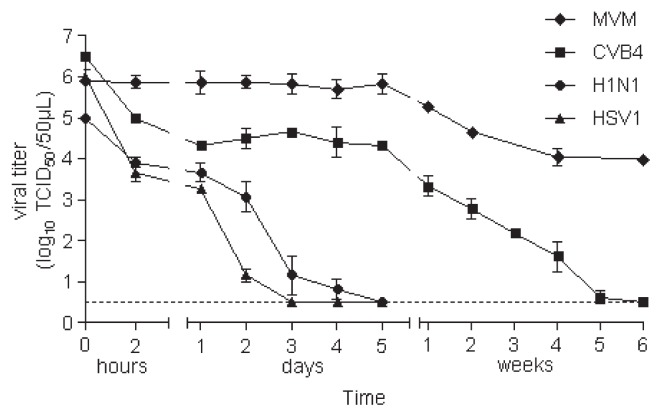
Virucidal effect of drying on viruses applied to Petri dish lids. Fifty microliters of each culture supernatant fluid containing H1N1, CVB4, HSV-1, or MVM was applied to Petri dish lids in quadruplicate. They were dried under the air flow of a biosafety cabinet at room temperature from 2 h to 6 weeks. Thereafter, dried inocula were recovered using 1 mL of titer media and the infectious titers were determined and expressed as log_10_. The results are the mean ± SD of four independent experiments. The dashed line represents the detection limit of the test.

Samples were considered dried when liquid was no longer observed on the lids, which occurred within 2 h. The mean values of the infectious titers of droplets containing HSV-1, H1N1, and CVB4 were reduced by 2.33 log_10_; 1.1 log_10_; 1.5 log_10_, respectively, 2 h after the inoculation (Fig. 1). Infectious viruses were not detected in dried HSV 1 and H1N1 inocula recovered 3 and 5 d after inoculation. In contrast, the viral titers in dried CVB4 inocula recovered 2 h to 5 d post inoculation were unchanged (4.39 +/− 0.38 log_10_ TCID_50_ mL^−1^, *n*=4). The viral titer of MVM in dried inocula recovered one week post inoculation was unchanged and similar to the titers measured in drops applied to the lids (5.87 +/− 0.20 log_10_ TCID_50_ mL^−1^ *n*=4). No infectious particle was detected in dried CVB4 inocula 6 weeks after inoculation, whereas a significant amount of infectious particles was recovered from dried MVM inocula (0.5 log_10_ TCID_50_ 50 μL^−1^ [detection limit] vs 4.00 log_10_ TCID_50_ 50 μL^−1^, respectively, *P* <0.02).

**Fig. 2:**
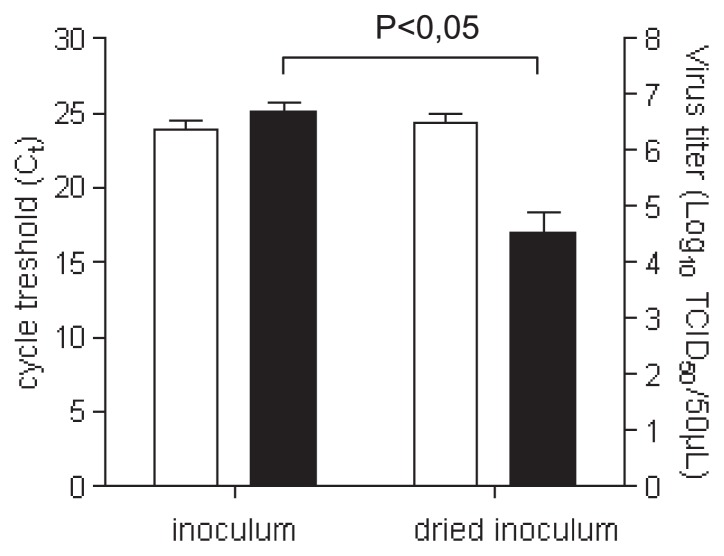
Quantification of CVB4 RNA and level of infectious particles. Fifty microliters of the culture supernatant fluid containing CVB4 was applied to petri dish lids in quadruplicate. Inocula on lids were dried for 2 h at room temperature, and, thereafter, recovered with 1 mL of culture media. The infectious titers were determined and expressed as log_10_ TCID_50_ 50 μL^−1^ (■). RNA was extracted and the levels of viral RNA were measured by quantitative RT-PCR and expressed as C_t_ (□). The results are the mean + SD of four independent experiments.

Reductions in the infectious titers in recovered inocula when drops were dried on petri dish lids raised several issues. The efficiency of recovering viral particles in these conditions was questioned. The infectious titer of CVB4 was determined to address this issue, and RNA was also extracted to measure the amount of viral RNA by RT real-time PCR in order to estimate the level of viral particles. The amounts of viral RNA in recovered inocula dried for 2 h at room temperature and in fresh inocula were similar, as displayed by the pattern of Ct values obtained by RT real-time PCR (mean values were 24.36 vs. 23.86, respectively, *P*=0.56), whereas the infectious titer values were markedly different (2.17 log_10_ reduction *P*=0.028) (Fig. 2). Taken together, these results demonstrated that viral particles dried on petri dish lids were readily recovered.

Thus, after drying on a plastic surface, the non-enveloped viruses, CVB4 and MVM, remained infectious for a longer period of time than the enveloped viruses, H1N1 and HSV-1.

### Repetitive cycles of drying and resuspension

The patterns of resistance of MVM, CVB4, H1N1, and HSV-1 were examined. Ten microliters of each clarified supernatant fluid containing MVM, CVB4, H1N1, and HSV-1 was dried as described above. Repetitive cycles of drying and resuspension were carried out: 10 μL of sterile distilled water was added to a dried spot to start a new cycle of drying. Thereafter, dried inocula were recovered using 1 mL of culture medium and the viral titers were determined.

**Fig. 3:**
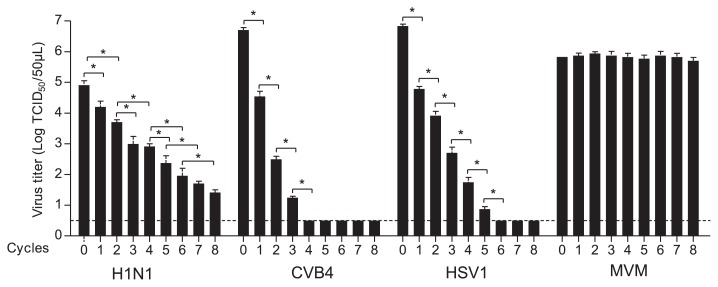
Virucidal effect of drying on H1N1, HSV-1, CVB4, and MVM in supernatant fluids applied to Petri dish lids. Ten microliters of each supernatant fluid was applied to Petri dish lids in quadruplicate. They were dried under the air flow of a biosafety cabinet at room temperature. Ten microliters of sterile distilled water was added to the dried spot before starting a new cycle. Thereafter, dried inocula were recovered using 1 mL of media and the infectious titers were determined and expressed as log_10_. The results are the mean + SD of four independent experiments. The dashed line represents the detection limit of the test. *: *P* value <0.05.

Each drying cycle resulted in a gradual reduction in the viral titers of H1N1, HSV-1, and CVB4 of approximately −0.4, −1.1, and −1.8 log_10_ TCID_50_ 10 μL^−1^ per cycle, respectively (Fig. 3). No reduction in the viral titer was detected for MVM after each cycle of drying. Furthermore, a reduction in viral titers was not observed in clarified supernatant fluids containing viruses kept at room temperature for the duration of the experiment (data not shown).

These results showed that iterative drying resulted in reductions in the viral titers of H1N1, CVB4, and HSV-1, whereas those of MVM remained unchanged.

### CVB4 dried in various media

Media containing various concentrations of FCS, BSA, and NaCl diluted in distilled sterile water were spiked with 1% of clarified CVB4-containing supernatants. Fifty microliters of these viral suspensions were applied to petri dish lids and dried, and viral titers in the recovered dried spots 2 h after the inoculation were determined.

**Fig. 4:**
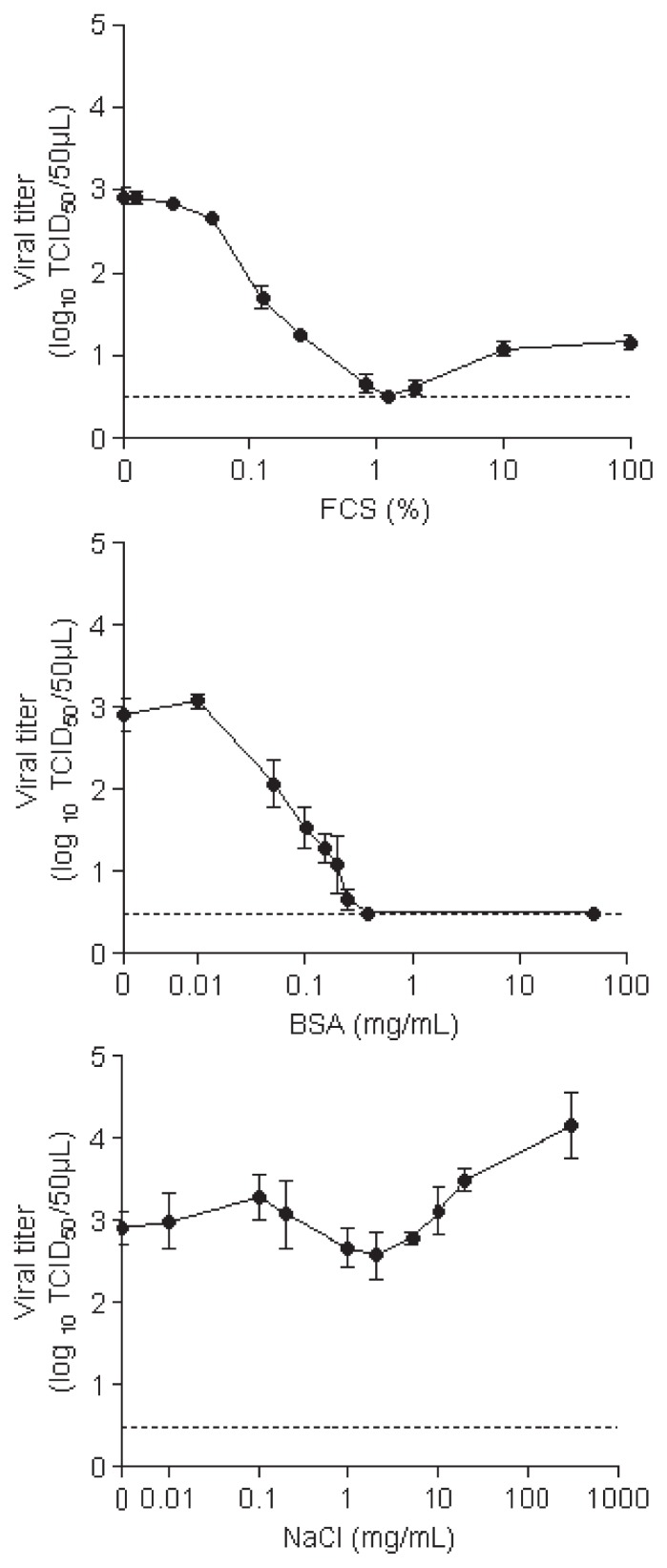
Virucidal effect of drying on CVB4 suspensions at various concentrations of FCS, BSA, and NaCl. Fifty microliters of a viral suspension was applied to Petri dishes in quadruplicate. They were dried under the air flow of a biosafety cabinet at room temperature. Thereafter, dried inocula were recovered using 1 mL of medium and the infectious titers were determined and expressed as log_10_. The results are the mean ± SD of four independent experiments. The dashed line represents the detection limit.

When the CVB4 suspension was spiked in medium containing a low concentration of FCS (<0.05%) or BSA (<0.01 mg mL^−1^) (Fig. 4), the viral titers in the recovered dried spots were higher at 2.66 and 3.08 log_10_ TCID_50_ 50 μL^−1^, respectively. In contrast, the viral titers in the suspension containing 1.25% of FCS and more than 0.39 mg mL^−1^ of BSA were below the detection limit of the test <0.5 log_10_ TCID_50_ 50 μL^−1^. The viral titers in the suspension containing more than 1.25% FCS increased steadily and reached 1.17 log_10_ TCID_50_ 50 μL^−1^ at 100% FCS (*P*=0.019 vs the detection limit of the test).

The viral titers in the recovered dried spots were lower when CVB4 was spiked in FCS and BSA media than in media containing any concentration of NaCl (Fig. 4). In media containing low concentrations of NaCl from 0 to 0.1 mg mL^−1^ the viral titers in the recovered dried spots increased slightly from 2.66 to 3.08 log_10_ TCID_50_ 50 μL^−1^ (*P*=0.1). In contrast, the viral titers in media containing between 0.1 and 2 mg mL^−1^ NaCl decreased from 3.08 to 2.58 log_10_ TCID_50_ 50 μL^−1^ (*P*=0.04). In media containing more than 2 mg mL^−1^ NaCl, the viral titers increased steadily and reached 4.16 log_10_ TCID_50_ 50 μL^−1^ at 300 mg mL^−1^ NaCl (*P*=0.028 vs. viral titers at 2 mg mL^−1^ NaCl).

**Fig. 5:**
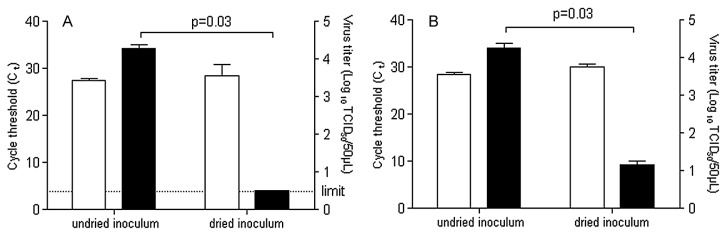
Recovery efficiency of CVB4 spiked in protein-rich medium. Culture supernatant fluid containing CVB4 was spiked (1% v/v) in medium containing BSA (50 mg mL^−1^) (A) and in FBS (100%) (B), and 50 μL of these suspensions were applied to petri dish lids in quadruplicate. Inocula on lids were dried for 2 h at room temperature, and, thereafter, recovered with 1 mL of culture media. The infectious titers were determined and expressed as log_10_ TCID_50_ 50 μL^−1^ (■). RNA was extracted and the levels of viral RNA were measured by quantitative RT-PCR and expressed as C_t_ (□). The results are the mean + SD of four independent experiments. The dashed line represents the detection limit.

The efficiency of recovering CVB4 when drops were dried in protein-rich media was analyzed. Viral titers were determined and RNA was extracted to measure the amount of viral RNA by RT real-time PCR. Experiments were performed with CVB4 spiked (1% vol/vol final dilution) in FBS (100%) or water containing 50 mg mL^−1^ BSA. In both protein-rich media, the amounts of viral RNA in recovered inocula dried for 2 h at room temperature and in undried inocula were similar, as displayed by the pattern of Ct values obtained by RT real-time PCR, whereas the infectious titer values were markedly different (Fig. 5). Taken together, these results demonstrated that viral particles contained in a protein-rich medium dried on petri dish lids were readily recovered.

## Discussion

Several considerations in the present study are noteworthy. The persistence of H1N1, HSV-1, MVM, and CVB4 was investigated and, for the first time, the effects of NaCl, FCS and BSA concentrations on CVB4 persistence were determined. Moreover, the effects of repetitive cycles of drying and resuspension on the persistence of H1N1, HSV-1, MVM and CVB4 were investigated. We selected petri dish lids for two reasons: they are hydrophobic, which prevents spreading of the droplet, and are non-porous, thereby improving virus viability (2). The efficiency of viral recovery from petri dish lids was confirmed in experiments combining the measurement of infectious titers and estimation of the levels of viral particles through the amount of viral RNA measured by RT real-time PCR.

Inactivation under the biosafety cabinet was achieved at different times based on the type of virus being tested. Inactivation curves for H1N1, HSV-1, and CVB4 encompassed two phases. The first phase lasted 2 h and corresponded to rapid decreases in the viral titer of −2.33, −1.1, and −1.5 log_10_ TCID_50_ mL^−1^ for HSV-1, H1N1, and CVB4, respectively, but not MVM. In the second phase, the viral titers of each virus slowly decreased. Scheuplein and al. reported that the first phase was characterized by water loss due to the evaporation of free water from the surface (14), which exposes viruses to a liquid-air interface, leading to virus inactivation (18). Repeated drying followed by resuspension in water was carried out for the first time in the present study. Infectious titers of H1N1, HSV-1, and CVB4 decreased gradually at each cycle, whereas no significant change was observed in the MVM infectious titer. Within these cycles, each resuspension modified particle positions and allowed for the new exposure of viral particles to the liquid-air interface, which may explain the continuous reductions observed in viral titers with each cycle (14). CVB4, which persisted for 5 weeks under the biosafety cabinet, was fully inactivated by 4 cycles of iterative drying, while H1N1 persisted for 5 d and remained infectious after 8 cycles. This difference can be explained by rehydration during the resuspension process, which markedly inactivated non-lipid viruses, as reported previously for poliovirus (3). However, MVM, a non-enveloped virus, was not affected by iterative drying.

Enveloped viruses were more sensitive than non-enveloped viruses in the second phase of viral persistence, which started when liquid was no longer observed on the lids; H1N1 and HSV-1 were inactivated in 5 d and 3 d, whereas CVB4 was inactivated in 6 weeks and MVM continued to be infectious. Drying was slower in this phase, which can be explained by the slow process of water loss due to the diffusion and elimination of bound water, as demonstrated previously (14). It remains to be determined whether the process is still slower in the case of MVM, as reflected by the persistence of this virus in our experiments.

A discrepancy was noted between our results and previous findings and was explained by several differences regarding media, surfaces, virus strains and temperature. Bean *et al.* inactivated H1N1 at a higher temperature (28.3°C vs. 20°C) (2) and Abad *et al.* inactivated poliovirus, an enterovirus resuspended in PBS, on a latex surface in less than 20 d (1) whereas CVB4 was inactivated in 6 weeks in our experiments.

The results of our study show that the initial concentrations of BSA, FCS, and NaCl played a role in the pattern of viability of CVB4 exposed to drying. The infectious titers of CVB4 after drying were higher when the virus was in pure water than in water containing BSA or FCS. These results demonstrated that the resistance of CVB4 to drying was hampered in the presence of BSA or FCS. The survival curves of CVB4 resuspended in 0 to 0.29 mg mL^−1^ BSA in 0 and 1.25% FCS were similar, which is consistent with the corresponding concentration of BSA in FCS under these conditions (0 and 0.30 mg mL^−1^). Taken together, these results suggest that the lower resistance of CVB4 in the presence of 0 to 1.25% FCS in our experiments depended on albumin. In contrast, with an initial concentration of more than 1.25% FCS, the impact of drying on CVB4 viability was reduced. A previous study reported that higher concentrations of proteins protected viruses against drying (16); however, CVB4 exposed to drying was fully inactivated in the presence of high concentrations of BSA (more than 0.39 mg mL^−1^) in the present study. The mechanism underlying the protective effect provided by high concentrations of FCS against drying remains to be determined. At an initial concentration of more than 10 mg L^−1^, NaCl protected CVB4 from desiccation. This can be explained by a slow rehydration process during sample recovery, which protected the viral capsid, as previously suggested (3). In contrast, the viability of CVB4 was the lowest at a concentration of 9 mg mL^−1^ NaCl, which is the average concentration in blood.

## Conclusion

We herein showed that viruses persisted for days or even weeks on dry hydrophobic surfaces. The pattern of resistance of viruses toward drying, as illustrated in the model of CVB4, was not due to the heterogeneity of viral populations, as suggested by the results of iterative drying. Moreover, media compositions and component concentrations clearly played a role when virus suspensions were exposed to drying. The results of our study suggest that a subsequent increase in solute concentrations in droplets modulated the viability of viruses toward drying. Since the compositions of media play a role in the viability of viruses exposed to drying, the persistence of viruses in natural media (clinical or environmental), instead of defined media, need to be investigated. Further studies will be directed towards this line of investigation in our laboratory.
